# Effect of Postural Control Demands on Early Visual Evoked Potentials during a Subjective Visual Vertical Perception Task in Adolescents with Idiopathic Scoliosis

**DOI:** 10.3389/fnhum.2017.00326

**Published:** 2017-06-30

**Authors:** Yi-Tzu Chang, Ling-Fu Meng, Chun-Ju Chang, Po-Liang Lai, Chi-Wen Lung, Jen-Suh Chern

**Affiliations:** ^1^Department of Educational Psychology and Counseling, National Taiwan Normal UniversityTaipei, Taiwan; ^2^Department of Occupational Therapy and Graduate Institute of Behavioral Science, School of Medicine, Chang Gung UniversityTaoyuan, Taiwan; ^3^Division of Occupational Therapy, Department of Rehabilitation, Chiayi Chang Gung Memorial HospitalChiayi, Taiwan; ^4^Graduate Institute of Medical Sciences, Chang Jung Christian UniversityTainan, Taiwan; ^5^Department of Medical Science Industry, Chang Jung Christian UniversityTainan, Taiwan; ^6^Department of Orthopaedic Surgery, Chang Gung Memorial Hospital, College of Medicine, Chang Jung UniversityTaoyuan, Taiwan; ^7^Department of Creative Product Design, Asia UniversityTaichung, Taiwan; ^8^Graduate Institute of Rehabilitation Counseling, National Taiwan Normal UniversityTaipei, Taiwan

**Keywords:** adolescent idiopathic scoliosis, subjective visual vertical, postural stability, event-related potentials, postural control

## Abstract

Subjective visual vertical (SVV) judgment and standing stability were separately investigated among patients with adolescent idiopathic scoliosis (AIS). Although, one study has investigated the central mechanism of stability control in the AIS population, the relationships between SVV, decreased standing stability, and AIS have never been investigated. Through event-related potentials (ERPs), the present study examined the effect of postural control demands (PDs) on AIS central mechanisms related to SVV judgment and standing stability to elucidate the time-serial stability control process. Thirteen AIS subjects (AIS group) and 13 age-matched adolescents (control group) aged 12–18 years were recruited. Each subject had to complete an SVV task (i.e., the modified rod-and-frame [mRAF] test) as a stimulus, with online electroencephalogram recording being performed in the following three standing postures: feet shoulder-width apart standing, feet together standing, and tandem standing. The behavioral performance in terms of postural stability (center of pressure excursion), SVV (accuracy and reaction time), and mRAF-locked ERPs (mean amplitude and peak latency of the P1, N1, and P2 components) was then compared between the AIS and control groups. In the behavioral domain, the results revealed that only the AIS group demonstrated a significantly accelerated SVV reaction time as the PDs increased. In the cerebral domain, significantly larger P2 mean amplitudes were observed during both feet shoulder-width-apart standing and feet together standing postures compared with during tandem standing. No group differences were noted in the cerebral domain. The results indicated that (1) during the dual-task paradigm, a differential behavioral strategy of accelerated SVV reaction time was observed in the AIS group only when the PDs increased and (2) the decrease in P2 mean amplitudes with the increase in the PD levels might be direct evidence of the competition for central processing attentional resources under the dual-task postural control paradigm.

## Introduction

Adolescent idiopathic scoliosis (AIS) is the most common type of spinal deformity among juveniles (Weinstein, 1994). Existing data indicate that AIS is characterized by decreased standing stability or poorer balance compared with normal age-matched teenagers (Gauchard et al., 2001; Nault et al., 2002; Guo et al., 2006; Beaulieu et al., 2009; Dalleau et al., 2011). Although, numerous etiologies, such as bilateral vestibular imbalance (Sahlstrand et al., 1979), bilateral paraspinal muscle imbalance (Ford et al., 1984), proprioception processing at the central level (Simoneau et al., 2006), and sensory integration disorder (Beaulieu et al., 2009), have been proposed for AIS, scientific understanding of the mechanism causing the poorer balance among AIS subjects remains vague. However, the possible central or peripheral etiologies imply that AIS subjects may demonstrate not only musculoskeletal impairment but also possible alteration of the sensory-perceptual processes that are managed by the central nervous system (CNS). Early in 1985, Herman et al. suggested that the vestibular signals interpreted by the CNS were highly correlated to the magnitude of the deviation associated with the curvature of the spine and may be an underlying factor for the high percentage of learning problems among AIS subjects. They further indicated that the presence of visuospatial perceptual impairment may be a common feature of idiopathic scoliosis. The authors hypothesized that learning deficits, altered visual/vestibular information processing and behavioral patterns, and scoliosis were interrelated. Until now, however, little attention has been paid to the role of the cerebral domain in the CNS, which might result in altered functional behavior after the occurrence or progression of scoliosis (Herman et al., 1985; Cheung et al., 2002; Beaulieu et al., 2009).

More recently, studies have proposed that the subjective visual vertical (SVV), which is the ability to visually perceive the earth vertically, is one type of visual information that is important for the regulation of static standing stability (Karnath et al., 2000; Karnath and Broetz, 2003; Bonan et al., 2006, 2007; Tarnutzer et al., 2009). Cheung et al. (2002) examined the SVV performance of adolescents with spinal deformity by instructing them to adjust a laser line projection to match the direction of gravity while standing. Equivalent accuracy of SVV performance was reported among AIS subjects, adolescents with congenital scoliosis, and age-matched controls. They therefore concluded that the perception of information modulating postural control in AIS subjects was not altered, and the cerebral domain impairment of postural control systems was not a cause of idiopathic scoliosis and its progression. However, deriving such conclusions from a single study seems ill-advised from our viewpoint. Due to the close link between SVV and postural control, previous studies have investigated SVV performance during various postural activities. For example, Bray et al. (2004) compared SVV performance levels in the postures of sitting, standing, or balancing on a beam. Their results showed that the SVV accuracy was better during unbalanced postures (on the beam) than balanced postures (sitting or standing); therefore, they concluded that “We are most aware of our place in the world when we are about to fall.” In line with Bray et al.'s finding (2004), Lopez et al. (2008) observed that the ipsilesional deviation of SVV judgment gradually increased from an upright standing position to sitting and lying supine positions among unilateral vestibular neurotomy patients during the first postoperative month. These findings suggest that the SVV performance is modulated by postural stability, and it is enhanced when postural control is more demanding. Because AIS populations are reported to have poorer postural stability than do adolescents without AIS, we hypothesized that the impact of postural demands on SVV judgment may be more crucial in the AIS population than in typically developed teenagers.

In the present study, to prove our hypothesis with neural-based evidence, we recorded online brain activities while the participants performed the SVV task in various standing postures. Event-related potential (ERP) was chosen as the most appropriate instrument for this study because it has been proven to have particular value for testing perception and attention, and it fit our experimental design with precise temporal resolution (Luck, 2005; Woodman, 2010). For exploring early sensory-perceptive processing through ERPs, the visual evoked potentials before 300 ms, such as P1, N1, and P2, are considered the most representative components associated with visual processing at the early stage. P1 is an occipital neural response that reflects the activity of the extra-striate area and fusiform gyrus between 85 and 130 ms (Luck, 2005; Di Russo et al., 2008). The subsequent N1 component is divided into an early N1 subcomponent that peaks around 100 to 150 ms at the anterior sites and a later N1 subcomponent that peaks around 150 to 200 ms at the posterior sites (Luck, 2005). Ungerleider and Haxby (1994) considered the N1 wave as the indicator of the reactivation of the striate and extra-striate areas through feedback from the higher visual areas and suggested that this re-efferent loop may be able to mediate the perceptual binding of stimulus attributes. Some studies have defined P1 and N1 as spatial attention directions (Herrmann and Knight, 2001; Hopf et al., 2002). Although, the former involves a large portion of the basic sensory processes, the latter mainly reflects the perceptive elements but not the cognitive processing of visually driven stimuli (Vogel and Luck, 2000; Herrmann and Knight, 2001; Hopf et al., 2002). Generally, both the P1 and the N1 components are sensitive to the direction of spatial attention (Mangun, 1995; Hillyard et al., 1998) and are modulated by selective attention (Luck et al., 2000). By contrast, P2 is a neural response elicited by fairly simple target features and requires a relatively lower cognitive loading than does P3 (Luck, 2005). Hence, because the materials for the SVV task used in this study were essentially simple and required comparatively minor cognitive or mental manipulation, we inferred that a P2 waveform, rather than the famous P3, would be elicited during the SVV processing. Moreover, as the functional meaning of the P2 component is closer to our concern of visual perception, we hypothesized that differential P2 performances might be observed between the AIS subjects and the age-matched controls. The early sensory processing of visual information was also examined through the inspection of the representative P1 and N1 components. Consequently, investigating the brain activities during the SVV task allowed for a rudimentary examination of the early sensory processing and visual perceptions in the AIS population and the influence of graded postural control demands (PDs); we then compared these data with those of the controls.

The present study investigated the effect of PDs on the SVV performances of the AIS and control groups to illuminate the cortical mechanism of postural control. This study also explored early sensory-perceptive processing in the CNS among the AIS subjects. The participants were asked to maintain their balance for as long as possible in different standing postures and to concurrently complete the SVV task, which resulted in a dual-task postural control condition (Bourlon et al., 2014). Online electroencephalograms (EEGs) and behavioral performances (the accuracy and reaction time [RT] for the SVV task, and center of pressure [CoP] excursions) were recorded. Hence, a comprehensive data set comprising the behavioral SVV performance, postural stability, and visual sensory-perceptual processing in the CNS (indexed by the stimulus-locked ERPs of the P1, N1, and P2 components) uncovered how PDs were interrelated to SVV processing in both the behavioral and cerebral domains. From the aforementioned literature review, we hypothesized that (1) the effects of PDs on SVV judgment would be more crucial for AIS populations than for typically developed teenagers and (2) differential P2 performances might be observed between the AIS group and the age-matched control group. The results of the present study inform preliminary arguments for the possible CNS pathogenesis of AIS.

## Methods

### Subjects

With our study design, a priori sample size calculation was made with statistical power of.8, alpha = 0.05, and beta = 0.02. The result showed that the number of participants in each group must be no <13 (Di Russo et al., 2008). Accordingly, 13 AIS subjects (3 males and 10 females; mean age, 15.65 years; range, 12–18 years; mean height, 1.61 m; range, 1.50–1.72 m; mean weight, 51.04 kg; range, 39–70 kg) were recruited from the orthopedic clinic of a medical center in northern Taiwan. Another thirteen healthy subjects, who were age-matched with the AIS group, were recruited from the same area as the control group (4 males and 9 females; mean age, 15.54 years; range, 12–18 years; mean height, 1.64 m; range, 1.50–1.77 m; mean weight, 52.85 kg; range, 40–70 kg). The age, height, and weight were not significantly different between the AIS and control groups. Both groups were comparable demographically. The inclusion criteria for the AIS group were as follows: (1) subjects who were diagnosed with AIS (Cobb angle more than 10°) by a pediatric orthopedic physician, (2) subjects who were aged between 12 and 18 years, (3) subjects who had received no active interventions before or at the time of entering the study, and (4) subjects who were without any severe neurological or pathological signs that might contaminate the results. The exclusion criteria for the AIS group were as follows: (1) subjects with a diagnosis of congenital scoliosis, neuromuscular scoliosis, or traumatic scoliosis; (2) subjects who had undergone spinal surgery before joining the study; (3) subjects diagnosed as having vestibular dysfunction; (4) subjects diagnosed with one or more of: mental retardation, attention deficit hyperactivity disorder, or autistic spectrum disorder; and (5) subjects with any other significant central or peripheral diseases that might contaminate the results. The inclusion and exclusion criteria were checked with and confirmed by the parents of the subjects who had volunteered to participate in this study.

All the subjects had normal or corrected-to-normal visual acuity and were right-dominant in their upper and lower limb use. Before the beginning of the experiment, all subjects were informed of the content and procedures to be performed. All subjects and the parents of the subjects gave written informed consent prior to taking part in the study. Each subject received a sum of NT$ 1,000 as compensation for participating in the experiment. The study was approved by the Chang Gung Medical Foundation Institutional Review Board.

### Stimuli

**Figure 1:**
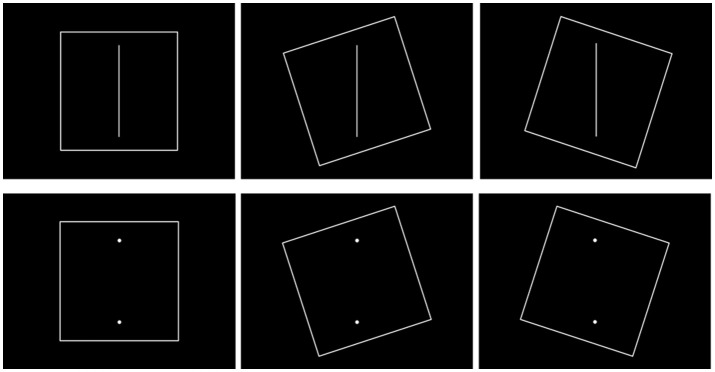
Examples of stimuli used during the mRAF test.

We modified the images outlined in the computerized rod-and-frame (mRAF) test proposed in Docherty and Bagust's study (2010) (Figure 1). The STIM II system (Compumedics Neuroscan, Australia) was used to deliver the SVV stimulus. The sequential file for displaying the mRAF test was tightly controlled using the Gentask Editor. All the stimuli were constructed using PhotoImpact 12.0 to ensure that specific angles were used, and the images were created at a high resolution of 1024 × 768 pixels. The frame and the visual rod were 0.2 cm in width, 50 cm and 40 cm in length, respectively. The background color was black. Three frame orientations were included: square untilted (0°), square tilted clockwise (+18°), and square tilted counterclockwise (−18°). The purpose of the frame was to create a perceptual bias. The following two rod formations were presented: a visible rod or two vertexes representing an invisible rod. The deviation degree of the rod was ±10° (+: clockwise; −: counterclockwise) with a 2° variation. Each angle of the rod was repeated two times. A total of 132 trials were subjectively judged by the participants in each required standing posture. All the stimuli were presented in a random order. It took 3–4 min to complete the mRAF test in each standing posture.

### Procedure

The major focus of the present study was to investigate the PDs that operated with the different shapes and different types of base support, using SVV performance as the assessment measure. Hence, all subjects were asked to complete the mRAF test in the following three standing postures in a random sequence: feet shoulder-width apart standing (D1), feet together standing (D2), and tandem standing (D3). The mRAF test was presented centrally using a 42-inch crystal screen placed at the subjects' eye level. Each stimulus was of 1,500 ms in duration, with a 500-ms interval between stimuli. Responses that occurred within 1,500 ms of stimulus presentation were considered valid responses.

Three practice blocks with six random stimuli were provided before formal measurement under the feet shoulder-width apart posture condition, to ensure that the subject was familiar with the mRAF test and operation of the response device. To complete the mRAF task, the subjects needed to assess if the rod was aligned with what they felt to be the true vertical. When the subject perceived the rod as being truly vertical, the subject clicked on the left button of the mouse, which was attached to the lateral side of the thigh ipsilateral to the dominant hand, with their index finger. When the subject perceived the rod as deviating from the true vertical, the subject clicked the right button with their middle finger. The instructions were as follows: “Keep your balance as much as possible and perform the mRAF task. It should take about 4–5 min to complete. Answer as quickly and accurately as you can.” Once the mRAF test had been initiated, the STIM II system automatically recorded the RT and whether the response was correct.

### Data recording

#### EEG

Continuous EEG was recorded using the Quik-Cap Electrode System and involved the measurement of digital data at 32 electric sites (A1, A2, FP1, FP2, F7, F3, FZ, F4, F8, FT7, FC3, FCZ, FC4, FT8, T7, C3, CZ, C4, T8, TP7, CP3, CPZ, CP4, TP8, P7, P3, PZ, P4, P8, O1, OZ, and O2); these were designated using a 10–20 electrode placement system, and Ag/AgCl electrodes were used. Two reference electrodes were positioned at the mastoid process of the ears. Ocular artifacts were monitored using bipolar pairs of electrodes positioned at the sub- and supraorbital ridges (vertical electrooculogram) and at the external ocular tail (horizontal electrooculogram). Electrode impedance was maintained below 10 kΩ. A Nuamps amplifier, together with Neuroscan 4.4 software (Compumedics Neuroscan, USA), was used to record and analyze the EEG signals continuously at a sampling rate of 1000 Hz by using the synchronous STIM II system.

#### CoP excursions

A RSscan foot pressure measurement system (Rsscan International Co., Belgium), which consisted of a 0.5-m pressure mat (578 × 418 × 12 mm) with high-intensity pressure sensors (a total of 4096 sensors arranged in a 64 × 64 matrix, with each active sensor area of ~4 cm^2^), and a three-dimensional processing unit, was used to record the two-dimensional CoP coordinates while the subjects were standing on the pressure mat in different postures. The CoP coordinates were exported to allow further analysis of the parameters representing the control of postural stability. A 100-Hz sampling rate was used during this study. The biomechanical measures of postural stability, behavioral performance of SVV, and ERPs were recorded simultaneously during each block of mRAF testing.

### Data analysis

#### ERPs

After the raw data had been acquired, offline analysis was performed using Neuroscan 4.4. First, manual artifact rejection was performed to discard eye movements or any other non-cerebral artifact. Second, the epoch was time-locked to the onset of each stimulus appearance, including a 100-ms prestimulus that served as the baseline and an 800-ms poststimulus. Only the epochs with the correct answer (an answer of “true vertical” for vertical, and an answer of “deviating from the true vertical” for non-vertical) were recruited for further analysis. Baseline correction was then conducted for all the remaining epochs, and they were corrected using the mean value of the electric potential before the trigger baseline. Trials contaminated by eye blinks or any other artifacts with voltage variations larger than ±100 μV at four EOG electrodes and ±80 μV at the resting electrodes were excluded. Before averaging, a band pass filter of 0.1–30 Hz was applied. Finally, at least 100 trials were retained for each posture condition. Average waves were separated by group and condition.

The focus of the analyses was the mean amplitudes and peak latencies (i.e., the time point at which the maximum positive or negative voltage occurred) of the three components, P1, N1, and P2, at different regions of interest. The time window for obtaining mean amplitudes and peak latencies was defined as 80–160 ms for the P1 component, 100–200 ms for the N1 component, and 200–300 ms for the P2 component. Measurements were conducted across the electrodes in which each component was maximal in amplitude in the grand averaged waveforms for each group. According to the literature review (Mangun, 1995; Hillyard et al., 1998; Luck, 2005) and the visual inspection of our data report, the mean amplitudes and peak latencies were defined as the averaged amplitude and averaged peak latency of O1, O2, and OZ electrodes for the P1 component; F3, F4, FZ, FCZ, and CZ electrodes for the N1 component; and P3, P4, and PZ electrodes for the P2 component. Visual inspections of the averaged data for each subject were performed to ensure that the ERP components were located within the respective time windows. Finally, the graphical electrophysiology data were outputted, converted into digital statistics, and categorized by group (AIS and control) and posture condition (D1: feet shoulder-width apart, D2: feet-together, and D3: tandem stance).

#### Behavioral performance

Behavioral performance included SVV performance and postural stability. The SVV performance was quantified in two ways. First, accuracy was calculated using the number of correct response trials divided by the total number of response trials. Second, RT was calculated as the average response time of all valid responses. The CoP coordinates recorded were exported to a custom-written MATLAB program (MathWorks, Inc, USA). The CoP excursion (mm) was the trajectory path length of the CoP in the transverse plane of each subject under each condition. It is the summation of the displacement of the CoP in the time window of the 132 trials. CoP excursion is a well-accepted measure of standing stability and is believed to indicate the volume of central commands used to regulate postural stability (Beaulieu et al., 2009). The CoP excursion datasets were normalized using body height because CoP excursion is considered a function of body height (Lin et al., 2010).

### Statistical analysis

For behavioral performance, two-way mixed-design repeated-measures analyses of variance (RMANOVA) were performed on the CoP excursion data, SVV accuracy data, and RT data. For electrophysiological data, single two-way ANOVA was used to evaluate the PD effects on the P1, N1, and P2 ERP components (mean amplitudes and peak latencies at different locations; occipital sites for the P1 components, frontocentral sites for the N1 components, and parietal sites for the P2 components). Greenhouse–Geisser adjustments to the degrees of freedom were applied to correct for the violation of the assumption of sphericity. Group (AIS group vs. control group) was defined as the between-subject factor, and static standing posture (D1: feet shoulder-width apart standing, vs. D2: feet together standing, vs. D3: tandem standing) was defined as the within-subject factor. Significant interactions were further investigated using the Bonferroni *post-hoc* analysis to demonstrate the simple main effects of either the standing posture or group. All statistics were considered significant at *p* < 0.05.

## Results

### Demographic characteristics

The Cobb angle in 9 of the 13 subjects in the AIS group was characterized by a right thoracic–left lumbar scoliosis curve, whereas the other four AIS subjects were characterized by a left thoracic–right lumbar scoliosis curve. Furthermore, 70% of the AIS subjects exhibited a double scoliotic curve (*n* = 9), with a further 23% exhibiting triple scoliotic curves (*n* = 3) and 7% exhibiting a single thoracic curve (*n* = 1). The average Cobb angle was 17.88° ± 8.29°, ranging between 7° and 40° at the thoracic spine (25.18° ± 9.44°) and between 14°and 39° at the thoracic–lumbar spine (Table 1). When considering the direction of the apex, the average Cobb angle at the thoracic spine was 15.5° ± 5.80° toward the left and 18.67° ± 9.05° toward the right. The average Cobb angle at the thoracic–lumbar spine was 25.25° ± 9.82° toward the left and 25.00° ± 10.39° toward the right.

**Table 1:** Average Cobb angle of the thoracic or thoracolumbar spine in the AIS group.

	**Averaged cobb angle**	**Cobb angle (L)**	**Cobb angle (R)**
Thoracic spine	17.88° ± 8.29°	15.5° ± 5.80°	18.67° ± 9.05°
Thoracic-lumbar spine	25.18° ± 9.44°	25.25° ± 9.82°	25.00° ± 10.39°

*Cobb angle (L): direction of apex toward the left side of the body*.

*Cobb angle (R): direction of apex toward the right side of the body*.

### Behavioral performance

#### Postural stability

No significant interaction was noted between the groups and postures (*F* = 0.33, *p* = 0.60). A significant main effect of posture (*F* = 107.01, *p* = 0.00) and a non-significant main effect of group (*F* = 0.35, *p* = 0.56) on postural stability were found. Multiple least significant difference comparisons revealed significant differences between the three standing postures (all *p* = 0.00). Both groups showed their largest excursion in the tandem stance and their smallest excursion in the feet shoulder-width apart stance. These findings indicate that the increase in CoP excursion was an effective indicator of the PDs across both groups (Table 2).

**Table 2:** Behavioral performance comparisons between the AIS and the control group.

	**CoP excursion (mm)**			**SVV accuracy (%)**			**SVV reaction time (ms)**		
	**AIS**	**Control**	***p*^1^**	**AIS**	**Control**	***p*^1^**	**AIS**	**Control**	***p*^1^**
**D1**	21.80 ± 5.92	22.63 ± 9.10	0.79	73 ± 18	77 ± 10	0.47	734.23 ± 163.96	630.82 ± 98.15	0.06
**D2**	47.53 ± 23.03	49.07 ± 15.95	0.84	76 ± 16	78 ± 10	0.75	638.10 ± 140.24	630.72 ± 126.80	0.89
**D3**	145.22 ± 48.71	159.65 ± 69.84	0.55	79 ± 15	79 ± 09	0.96	579.40 ± 119.45	617.18 ± 118.52	0.43
***p***^2^	0.00^**^	0.00^**^		0.19	0.11		0.00^**^	0.89	

*D1: feet shoulder-width apart, D2: feet together, D3: tandem stance*.

p^1^: group main effect, p^2^: posture main effect, p < 0.01^**^.

*Values are expressed as mean ± SD. For CoP excursion, both the AIS and control groups demonstrated the largest excursion in the tandem stance, followed by the feet together stance, and the smallest excursion in the feet shoulder-width apart stance. For SVV accuracy, both the AIS and control groups demonstrated equivalent SVV accuracy, regardless of the standing posture. For SVV reaction time (RT), the AIS group demonstrated the quickest RT in the tandem stance, followed by the feet together stance, and slowest RT in the feet shoulder-width apart stance. The control group demonstrated equivalent RTs, regardless of the standing posture*.

#### SVV accuracy

Statistical analysis revealed neither a significant interaction between group and posture (*F* =.710, *p* = 0.438) nor a main effect of posture (*F* = 3.188, *p* = 0.08) or group (*F* = 0.17, *p* = 0.69) on SVV accuracy. The SVV accuracy of the AIS group was comparable to that of the controls, regardless of the standing posture (Table 2).

#### SVV RT

The results showed a significant interaction between the groups and postures (*F* = 5.25, *p* = 0.01) with respect to RT. Subsequent Bonferroni analysis revealed a significant simple main effect of posture in the AIS group (*F* = 13.539, *p* = 0.01) but not in the control group (*F* = 0.12, *p* = 0.89). No simple main effect of group was noted for any of the stance postures (all *p* > 0.05). *Post-hoc* analysis indicated that the AIS group demonstrated the quickest RT in the tandem standing posture, followed by the feet together standing posture, and the slowest RT was in the feet shoulder-width apart standing posture (all *p* = 0.00). The RTs of the controls were similar across all standing postures (Table 2).

### ERPs

**Figure 2:**
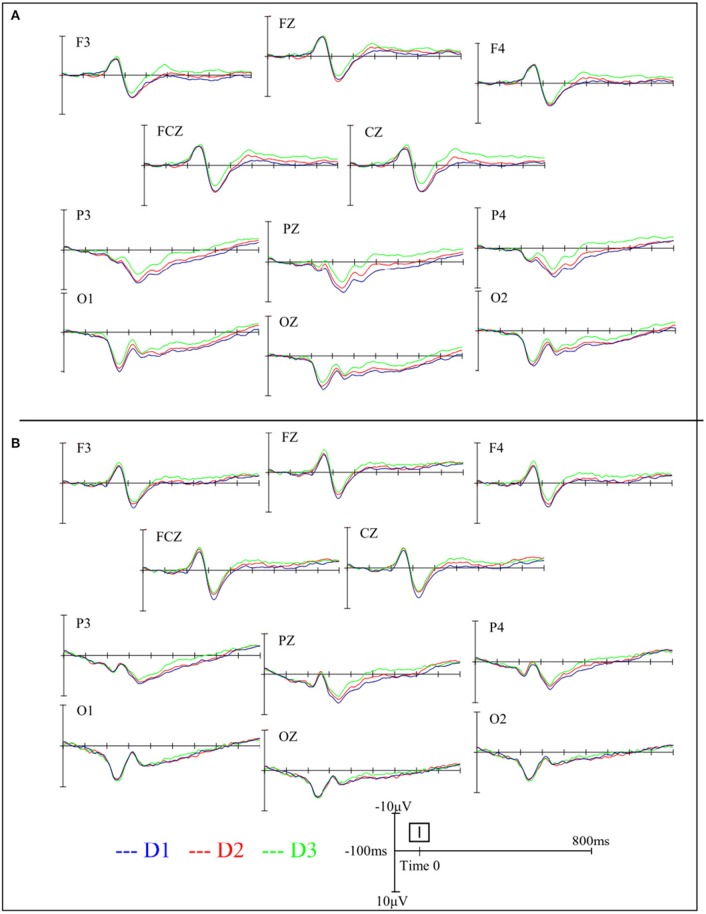
Grand average waveforms in the AIS group **(A)** and the control group **(B)** during different standing postures at various electrode sites.

**Figure 3:**
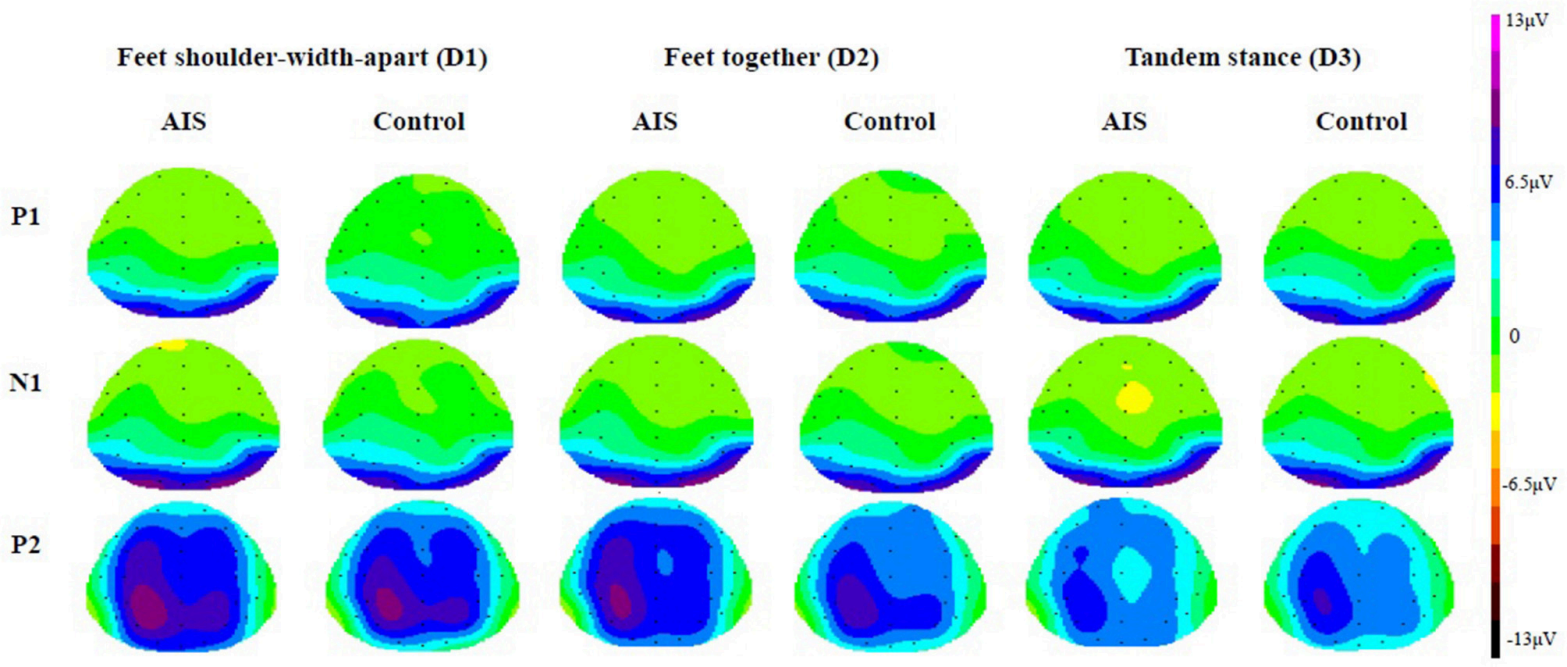
Grand average topographies in the AIS group and the control group (View from top).

Figures 2, 3 depict the grand average waveforms and topographies for the AIS and control groups during different standing postures. For peak latencies, two-way ANOVAs demonstrated neither any interaction effect nor main effect of group or posture on the P1, N1, and P2 components (all *p* > 0.05). For the mean amplitudes of the P1 component at occipital sites, statistical results revealed neither any interaction effect nor main effect of group or posture. For the mean amplitudes of the N1 component at the frontocentral sites, neither any interaction effect nor main effect of group was noted, but statistical results revealed the main effect of posture (*F* = 3.41, *p* = 0.04). However, *post-hoc* Bonferroni analysis indicated no significant difference between the postures (all *p* > 0.05). Finally, for the mean amplitudes of the P2 component at parietal sites, neither any interaction effect nor main effect of group was noted, but statistical results revealed the main effect of posture (*F* = 14.39, *p* < 0.01). Subsequent *post-hoc* Bonferroni analysis indicated that both the feet shoulder-width apart standing (mean ± SD = 6.47 ± 0.78 μV) and feet together standing (mean ± SD = 5.78 ± 0.67 μV) postures demonstrated significantly larger P2 mean amplitudes than did the tandem standing posture (mean ± SD = 4.23 ± 0.62 μV; all *p* < 0.01). All numerical data of the mean amplitudes and mean peak latencies of the P1, N1, and P2 components in the AIS and control groups are listed in Table 3.

**Table 3:** Mean amplitudes and mean peak latencies of the P1, N1, and P2 components in the AIS control group.

	**Mean amplitude (μV)**		**Peak latencies (ms)**	
	**AIS**	**Control**	**AIS**	**Control**
**P1 (80–160 ms)**				
D1	5.15 ± 3.17	4.95 ± 2.87	143.56 ± 17.22	151.67 ± 13.98
D2	4.98 ± 3.06	5.01 ± 3.26	149.72 ± 18.85	150.36 ± 17.24
D3	4.63 ± 3.37	5.14 ± 2.90	143.40 ± 15.58	149.72 ± 13.41
**N1 (100–200 ms)**				
D1	−2.52 ± 1.73	−1.52 ± 2.08	156.71 ± 10.60	147.89 ± 13.03
D2	−2.19 ± 2.29	−2.02 ± 2.19	154.26 ± 11.60	151.22 ± 14.05
D3	−3.10 ± 2.09	−2.51 ± 2.56	157.28 ± 11.24	154.94 ± 12.22
**P2 (200–300 ms)**				
D1	6.57 ± 3.67	6.36 ± 3.67	244.15 ± 17.67	250.10 ± 15.88
D2	5.80 ± 2.92	5.75 ± 3.61	236.54 ± 20.28	246.18 ± 15.02
D3	4.07 ± 2.90	4.40 ± 3.15	238.64 ± 23.67	242.44 ± 17.54

*D1: feet shoulder-width apart, D2: feet together, D3: tandem stance*.

*Values are expressed as mean ± SD. The mean amplitudes and peak latencies are the average of mean amplitudes and peak latencies of O1, OZ, and O2 electrodes for the P1 component; F3, FZ, F4, FCZ, and CZ electrodes for the N1 component; and P3, PZ, and P4 electrodes for the P2 component. Significance was only reported for the main effect of posture for the mean amplitudes of the P2 component (F = 14.39, p < 0.01). Subsequent post-hoc Bonferroni analysis indicated that both D1 and D2 postures demonstrated significantly larger P2 mean amplitudes than D3 did. None of the rest results revealed any interaction effect or main effects of posture or group*.

## Discussion

The present study is a prospective pilot study that investigated the interaction of postural stability with SVV performance among adolescents with and without idiopathic scoliosis. More specifically, we examined the effect of PDs on visual vertical perception among these subjects, using the ERP approach. First, our results revealed that both adolescents with or without AIS demonstrated increased CoP excursions and decreased P2 mean amplitudes as the PDs increased, elaborating the general performance of the competition for the limited central resources between the postural task and the concurrent SVV task across these subjects, which is in accordance with the cross-domain competition model derived from the dual-task paradigm (Andersson et al., 2002; Pellecchia, 2003; Lacour et al., 2008; Palluel et al., 2010). Second, inconsistent with previous studies' results that AIS subjects have poorer postural stability or balance performance (Gauchard et al., 2001; Nault et al., 2002; Guo et al., 2006; Beaulieu et al., 2009; Dalleau et al., 2011), we observed competitive postural stability under the concurrent measurement of the SVV task, as well as the same SVV accuracy, among the AIS subjects compared with the controls. Nevertheless, SVV RT gradually accelerated as the PDs increased, but this was observed in the AIS group only. By contrast, the level of PDs had no effects on concurrent SVV task performance in the controls. The present study used the ERP approach to preliminarily investigate the early sensory-perceptive processing and the influence of PDs and to compare these data between the AIS subjects and controls. However, no group, PD interference, or AIS main effects on the mean amplitudes and peak latencies of the P1, N1, and P2 components were noted. Despite the lack of any significant pairwise difference, the tendency of the PD effects on the N1 mean amplitude is noteworthy and supports our hypothesis that postural control demands attentional resources, especially when the PDs increased. The lack of significant differences among the stance postures might be due to insufficient cognitive demands imposed by the mRAF task used in this study. Thus, the findings of the present study partially confirm our hypothesis that the interference of PDs in SVV performance with respect to the behavioral domain is more crucial in the AIS population than in age-matched teenagers. Our results failed to support the hypothesis of potentially different P2 performance levels in the cerebral domain between the AIS subjects and controls. We highlight that a differential behavioral strategy might be adopted for the weighting and allocation of attention resources while concurrently coping with PDs and SVV processing in the AIS group.

### Behavioral performance and a reorganized behavioral pattern among AIS subjects

Unlike previous studies that manipulated the difficulty across the cognitive tasks and examined the impact of varied cognitive loads on postural control (Andersson et al., 2002; Pellecchia, 2003), the present study provides the opposite insight by manipulating the PDs across the postural tasks to examine the effect on concurrent visual perception; this contributes to the uniqueness of the present study in the research field of dual-task conditions. Seemingly, the observed comparable CoP excursions in each standing posture between the groups disproved the established proposal of a poorer standing stability in subjects with AIS than in normal age-matched adolescents (Gauchard et al., 2001; Nault et al., 2002; Guo et al., 2006; Beaulieu et al., 2009; Dalleau et al., 2011). However, we speculate the differences in the results for the standing stability of AIS subjects between the present and other studies might be due to the different contexts in which the postural stability was measured. In previous studies, the postural stability was measured while simply requiring the subjects to stand as stably as possible, without the need to pay attention to any concurrent visual task, whereas in our study, the standing stability was measured during concurrent active visual tasks (the SVV task). In a more recently published review article, significantly lower amplitude of the body displacement was reported under active visual tasks than under the control quiet stance task (Bonnet and Baudry, 2016). Although speculative, we wonder whether this may be the helping effect of the concurrent visual vertical perception processing that contributes to the comparable postural stability between AIS subjects and controls. Additional evidence is needed to clarify whether the SVV acts as a profound helping effect during the regulation of the postural stability among AIS individuals. Another factor contributing to the similar postural stability performances between the groups might be the insensitive parameter of CoP excursions used in this study.

A gradual increase in SVV RT with the increase in PDs was observed in the AIS group but not in the control group, indicating the greater influence of PDs on SVV performance in AIS individuals. We suggest that a reorganized behavioral pattern is present in AIS subjects such that when the PDs increased, the subjects speed up their completion for the concurrent task. Because no related research has examined the behavioral performance of AIS subjects during cognitive or perceptive tasks, the underlying mechanism leading to the acceleration strategy that is noted in AIS subjects when faced with dual-task conditions needs to be further explored. Currently, we infer that the increased speed for the SVV task among the AIS subjects tends to hasten the completion of the SVV task, the central commands are rapidly reallocated, and postural stability is regained; otherwise, these subjects would either fall or fail to complete the SVV task. Of note, the tandem stance used in this study is a challenging standing posture for both groups. However, the influence of the tandem stance is not as threatening to the controls because they are seemingly able to coordinate their limited central resources between the SVV task and even the most challenging tandem stance. By contrast, the threatening influence of the tandem stance might become harmful to the AIS subjects due to their potentially poorer balance control. Accordingly, when task integration becomes maximally challenging as the attention demands of the postural tasks increase to the highest level but remain affordable to the AIS subjects (indexed by the comparable CoP excursion and SVV accuracy), they tend to adopt the accelerated strategy to successfully complete the SVV task as requested.

### Similar cortical responses when processing PDS and SVV simultaneously between the AIS subjects and controls

The ERP results showed that the early sensory responses driven by visual SVV stimuli are similar in both groups, and there is a lack of significant differences with respect to the P1 and N1 components. Our finding indicates that when PDs are increased, both the AIS and control groups show decreased P2 mean amplitudes at parietal sites, suggesting that when PDs are increased, a common feature of allocation of the mental resources for the control of postural stability and the judgment of SVV is activated regardless of the spinal deformity. This phenomenon is in line with the competitive sharing of attentional resources or dual relations, such as the generated models from cognitive approach (Bourlon et al., 2014; Jehu et al., 2015). Little and Woollacott (2015) reported attenuated N1 peak amplitudes in dual-task conditions (a visual working memory task paired with a postural task) compared with the single-task conditions, and they therefore concluded that the N1 ERP component might support the competitional theory between the two tasks, with diverted attentional resources from the processing of sensory inputs being associated with the postural perturbation (Dietz et al., 1984; Quant et al., 2004). By contrast, our results demonstrate a reduction in the P2 mean amplitudes of the ERP as the PDs increased, and we suggest P2 mean amplitudes as important direct evidence of the competition for central processing attentional resources under the dual-task postural control paradigm. The differences in findings might be due to the use of graded PDs and the different secondary tasks used in our study and in Little and Wollocott's study. The ERP index revealing cortical involvement during dual-task postural control might be specific to PDs or cognitive tasks. A more deliberate research design and inferences are needed for insights into the interaction between postural and concurrent cognitive or perceptive tasks, especially from a neural perspective.

The PDs mainly seemed to affect the mean amplitude of the P2 component at the parietal lobe of the AIS subjects, which can be explained based on existing findings suggesting that the parietal lobe is a polymodal sensory area that not only integrates multiple vertical forms of perception, including visual, vestibular, and somatic sensory information, and somatic feedback (Bonan et al., 2006; P'erennou et al., 2008) but also plays an important role as the control center for postural control, a prerequisite for successful postural stability (Lalonde and Strazielle, 2007; Shumway-Cook and Woollacott, 2007). This implies that the parietal lobe has at least two simultaneous roles, processing postural control and integrating multiple vertical perceptions. Bonan et al. (2006) and Karnath et al. (2000) have also proposed that the vestibular cortex, which includes the posterior insula, superior temporal lobe, middle temporal lobe, and inferior parietal lobe, has a dominant, though not exclusive, role in visuospatial perception. This polymodal sensory area is known to respond to either visual or vestibular inputs that are associated with somatic sensory processing in relation to gravity (P'erennou et al., 2008); furthermore, damage to this system is significantly correlated with the balance performance after stroke (Bonan et al., 2006). In addition, parietal lobe impairment leads to subjective visual, postural, and haptic vertical perception deviations (P'erennou et al., 2008), which confirms the importance of the parietal lobe to the multiple model and infinite internal models of vertical perception.

Finally, the hypothesis that subjects with and without AIS might possess different cortical mechanisms during dual-task postural control, especially when the PDs are increased, was not fully supported by our results. This might have been due to the inadequate interference of the cognitive task in the cortical mechanism for postural control measured by ERPs used in this study, suggesting that the AIS subjects could maintain postural stability comparable to that of controls by using automatic processes, without the need to devote additional attention. However, the observations that the CoP excursion in AIS subjects tended to be larger (although without group significance) than that in controls and that the levels of PDs influenced the SVV performance, specifically the SVV RT, indicated that the automatic process for postural control in AIS might not be as effective as that in controls. The other reason might be that the AIS subjects recruited in this study were all characterized by double curves of S shape. The adverse effects of the thoracic curve on postural stability might be compensated by the lumbar curve (Kuo et al., 2010; Nowotny et al., 2012). The posture in AIS subjects observed in this study was not as unstable as expected. We, therefore, hypothesize that adaptive postural control strategies and sensory-perceptive processes might have occurred in subjects who participated in this study. This is a preliminary study investigating the interrelations among AIS, PDs, and SVV. The proposed hypotheses warrant further investigation.

### Recommendations for future studies

AIS has been considered as an orthopedic disorder. Therefore, clinical and rehabilitative interventions mainly focused on the biomechanical domain, such as surgical correction or wearing a brace to reconstruct the deformed spinal alignment. The subjects participating in this study had never undergone any form of spinal correction. However, through the dual-task paradigm, the present study provided preliminary evidence of behavioral reorganization in AIS subjects. In reality, most daily tasks are basically dual tasks. Thus, it is uncertain whether the behavioral performance levels of AIS subjects remain competitive as the cognitive loads of secondary tasks increase prominently. Therefore, the present study reaffirms the importance of comprehensively exploring behavioral performance and its possible influences on the daily performance levels of AIS subjects. Thus, improved forms of clinical assessments and rehabilitative guidelines for the AIS population are likely to be developed.

The main limitations of the present study were the small sample size and the highly homogenous spinal pathological characteristics of the AIS subjects. Increasing the number of AIS subjects and classifying them into subgroups according to curve numbers, angles, and locations might help to clarify the effects of the types and severity levels of spinal deformation on SVV and postural stability, which can facilitate investigation of the associated cortical mechanisms for postural control.

## Conclusions

The present study is a preliminary effort to examine the central mechanism involved in static standing stability and SVV in an AIS population. The findings indicate that the PD effect was more crucial in the AIS subjects. During the dual-task paradigm, a differential behavioral strategy was adopted by AIS subjects when the PDs increased, whereas the PDs had no effect in the controls. Despite the lack of significant group differences in the cerebral domain, our results indexed the reduction in the P2 mean amplitudes as the PDs increased, which is important direct evidence of the competition for central processing attentional resources between the dual tasks in this study. The importance of providing insights into the comprehensive performance levels in terms of behavioral or neural aspects among AIS subjects was reaffirmed by the present study.

## Author contributions

YC contributed to the study design, experiment setup, data acquisition and processing, statistical analysis, and manuscript drafting. LM contributed to the conceptual work of the ERP design, provided neurological advice, helped to explain the results, and helped to draft the manuscript. CC contributed to data interpretation, data processing, and manuscript drafting; PL contributed to subject recruitment, provided medical advice, and helped to design the conceptual aspects of the study. CL contributed to the data analysis and programming. JC contributed to the innovativeness of the research idea, the conceptual work of dual-task postural control, the design of the study, and the IRB processing; JC was in charge of raising funds for the project, aided with data interpretation, helped to critically revise the manuscript, and was involved in the final approval of the published version. All the authors agree to be accountable for all aspects of the work and to ensure that questions related to the accuracy or integrity of any part of the work are appropriately investigated and resolved.

### Conflict of interest statement

The authors declare that the research was conducted in the absence of any commercial or financial relationships that could be construed as a potential conflict of interest.
